# Normalization of array-CGH data: influence of copy number imbalances

**DOI:** 10.1186/1471-2164-8-382

**Published:** 2007-10-22

**Authors:** Johan Staaf, Göran Jönsson, Markus Ringnér, Johan Vallon-Christersson

**Affiliations:** 1 Division of Oncology, Department of Clinical Sciences, Lund University, 221 85 Lund, Sweden

## Abstract

**Background:**

High-resolution microarray-based comparative genomic hybridization (CGH) techniques have successfully been applied to study copy number imbalances in a number of settings such as the analysis of cancer genomes. For normalization of array-CGH data, methods initially developed for gene expression microarray analysis have, in general, been directly adopted and used. However, these methods are designed to work under assumptions that may not be valid for array-CGH data when copy number imbalances are present. We therefore sought to investigate the effect on normalization imposed by copy number imbalances.

**Results:**

Here we demonstrate that copy number imbalances correlate with intensity in array-CGH data thereby causing problems for conventional normalization methods. We propose a strategy to circumvent these problems by taking copy number imbalances into account during normalization, and we test the proposed strategy using several data sets from the analysis of cancer genomes. In addition, we show how the strategy can be applied to conveniently define adaptive sample-specific boundaries between balanced copy number, losses, and gains to facilitate management of variation in tissue heterogeneity when calling copy number changes.

**Conclusion:**

We highlight the importance of considering copy number imbalances during normalization of array-CGH data, and show how failure to do so can deleteriously affect data and hamper interpretation.

## Background

Microarray-based techniques for genome-wide investigation of copy number aberrations (CNAs) have recently gained much attention. Initially employing arrays developed for gene expression analysis [1], or low-density arrays produced from large-insert genomic clones such as bacterial artificial chromosomes (BACs) [2], the application has evolved rapidly. Currently, specialized high-density arrays with oligonucleotide probes or probes derived from BAC clones are predominately used. Two-channel array-based comparative genomic hybridization (aCGH) is a direct successor to conventional metaphase CGH [3]. In both cases, DNA from two samples are differentially labeled with fluorescent dyes and co-hybridized to immobilized genomic capture probes. By use of aCGH, DNA derived from tumor tissue can be compared with reference DNA, e.g., normal whole blood DNA, and genomic imbalances can effectively be investigated. The main advantage of aCGH over conventional CGH is the increased resolution achieved by microarrays with a large number of individual probes, routinely up to hundreds of thousands, covering the entire genome [4]. The power of aCGH has been demonstrated in tumor studies [5-8], as well as in the field of clinical genetics [9], and the basis of the technique is reviewed elsewhere [10]. In essence, relative ratios of copy number between two DNA samples are obtained by comparing the two fluorescent signal intensities for each probe under the assumption that intensities reflect the amount of corresponding genomic DNA in the respective sample.

**Figure 1:**
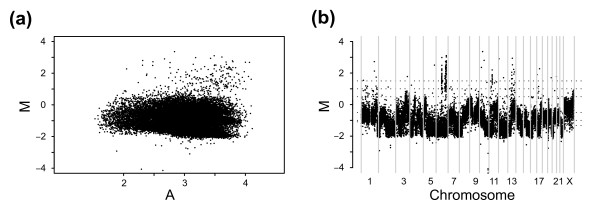
Genome plot and M-A plot representing two frequently used ways of visualizing aCGH data. Plots show data from the L56Br-C1 xenograft [19] analyzed using a tiling 32 K BAC array and illustrate how copy number imbalances readily observed in genome plots can be difficult to discern as copy number populations in M-A plots. **(a) **M-A plot of un-normalized data. **(b) **Genome plot of un-normalized data.

In much the same way as for gene expression microarray analysis, relative ratios must be normalized to account for systemic technical bias while retaining relevant biological changes [11]. Although much effort has been invested in developing methods for analysis of aCGH data, including break-point identification and segmentation [12-14], less attention has been devoted to normalization. For this latter purpose, methods originally developed for gene expression microarray data, such as global-median (Median) and intensity-based lowess (Lowess) normalization, have been adopted [5,6]. Recent reports have evaluated the performance of gene expression normalization strategies when applied on aCGH data and have proposed more specific approaches [15,16]. Although valid concerns about directly adopting existing normalization techniques are expressed, proposed strategies rely on available conventional methods and the inherent properties of aCGH data have, rather than being incorporated in the strategies, mainly been used for calibration and validation. Microarray data is frequently visualized using M-A plots in which the log ratio, referred to as M, is plotted as a function of log mean intensity, referred to as A (Figure 1a) [17]. When normalizing data using Median, the median M value is identified and subtracted from all M values. This procedure centers data such that the median M value becomes zero. Lowess normalization works in much the same way but use a locally fitted regression curve along the full range of A to identify M values to center data at. This intensity-based strategy has the added advantage over Median normalization of correcting for intensity-based bias of M. Intensity-based bias can introduce curvature in M across A (Figure 1a) which remains uncorrected for after Median based normalization.

Using self-self comparisons, in which a sample is compared with itself, it has been observed that other forms of technical bias, e.g., spatial- or plate bias, exist that can skew measured M values enough to revoke the validity of the aforementioned normalization methods [17]. Both methods have therefore been implemented in ways that include stratification of M values in groups of data that are individually subjected to the correction. Stratification can be performed based on, e.g., spatial probe location, or probe source [17]. The general thought is that stratification will result in groups, i.e., populations, of data in which the validity of the normalization method is upheld. It has also been observed that the assumptions, required for conventional normalization methods to work, can fail as a result of a true biological distribution of M, e.g., in situations where the majority of probes measure true differences between compared samples [18].

We here highlight a well known and commonly displayed property of tumor cells, namely the presence of biologically true CNAs. Figure 1b shows a genome plot of raw M values obtained by aCGH of a female breast cancer tumor xenograft [19] compared with male normal whole blood DNA. In the genome plot, M is plotted as a function of the genomic location of the probe sequence. In figure 1b, several genomic regions with different and discrete M can readily be observed. We sought to investigate the effect on normalization imposed by this property of aCGH data. We show that this property results in consequential drawbacks when using conventional normalization methods and propose a strategy that incorporates any populations present in the data into the normalization.

The proposed strategy can be integrated with any of several existing normalization methods and results in improved data quality. Also, spatial effects resulting in non-biological, but relevant, populations that can bias normalization are handled when calculating corrections. We also note that part of the procedure can be applied to assign adaptive sample-specific thresholds for calling copy number changes. The proposed normalization strategy, as well as the adaptive sample-specific level scaling, provides powerful and convenient means for improved copy number analysis using aCGH.

## Results and Discussion

This study is outlined as follows with results and discussion presented accordingly. To investigate the influence of copy number imbalances on normalization we first created a set of mimicked data representing states of an increasing fraction of genomic gain. Using the mimicked data we demonstrate the effects of gain on normalization using Median and Lowess. We then evaluated an alternative normalization strategy in which data is stratified into separate populations representing gain and balanced copy number respectively. Whereas mimicked data provide prior knowledge facilitating stratification, most experiments lack this information. Therefore, we developed a method for stratification of data and evaluated the method using previously characterized cases. By applying our procedure for stratification and normalization to tumor specimens on different aCGH platforms we compare performance with standard methods. We investigate the implication of technical spatial effects and propose a strategy for improved normalization. In addition, we evaluate the possibility to apply our method to assess noise levels in data and assign sample-specific thresholds for detection of copy number imbalances.

### Normalization of aCGH data using Median

**Figure 2:**
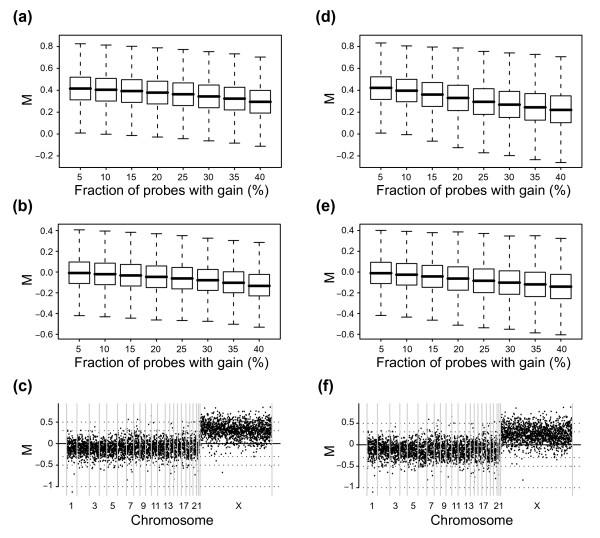
Median and Lowess normalization of aCGH data. Data is from a normal female with the karyotype 46, XX and a cell line with 47, XXX. Data sets with mimicked fractions of probes with gain (5, 10, 15, 20, 25, 30, 35, or 40 percent) were constructed by randomly omitting varying number of probes for autosomes. Box-plots display M values after normalization for data sets with varying fraction of probes with gain. **(a) **M values for X-chromosome probes after median normalization. **(b) **M values for autosomal probes after median normalization. **(c) **Genome plot after median normalization for the data with 35 percent of probes with gain. **(d) **M values for X-chromosome probes after lowess normalization. **(e) **M values for autosomal probes after lowess normalization. **(f) **Genome plot after lowess normalization for the data with 35 percent of probes with gain.

We assumed that aCGH data from samples with a substantial amount of imbalances could be erroneously corrected using Median normalization. This problem is not unexpected and the effect is well known in corresponding cases when gene expression microarray data is normalized [18]. We investigated this issue using aCGH data derived from tiling BAC arrays comparing copy number between DNA from a normal female with karyotype 46, XX and a cell line with 47, XXX [20]. In this case, autosomes are expected to yield log ratio values of M = 0 and the X chromosome is expected to yield log ratio values of M = 0.58 corresponding to XXX/XX. By first removing Y chromosome values and then randomly omitting a varying number of values for autosomes, while retaining all X chromosome values, we could mimic cases with different percentage of gain. In this way we created mimicked data sets with 5, 10, 15, 20, 25, 30, 35, and 40 percent gain, respectively, where 5 percent gain corresponds to not omitting any autosome values. Data sets were created from raw data and then subjected to normalization using Median. After normalization we investigated ratios for autosomes and the X chromosome (Figure 2). As a result of an increased fraction of gain, the median M for the X chromosome is shifted from 0.42 to 0.30 (Figure 2a), confirming our belief that normalization strategies for aCGH should account for the presence of different copy-number populations. The observed shift is a direct result of the composition of aCGH data with respect to copy number populations and can also be observed when looking at autosomes for which the median M is shifted from -0.01 to -0.13 (Figure 2b). When visualizing the normalized data in genome plots the shift clearly appears: M = 0 is in between the two populations (Figure 2c).

### Genomic imbalances correlate with intensity in aCGH data

**Figure 3:**
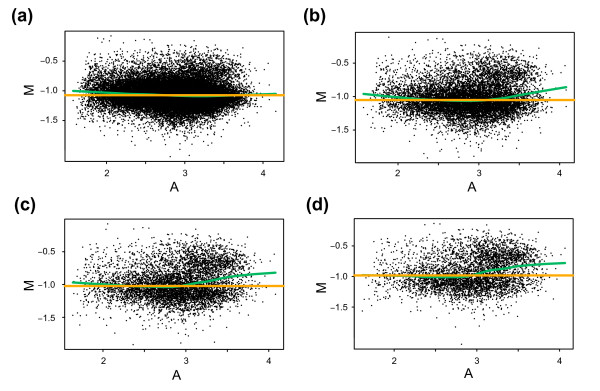
Differences between Median and Lowess normalization. M-A plots of un-normalized log ratios with correction lines for Median (orange) and Lowess (green) normalization. The plots show data from figure 2 for data sets with mimicked fraction of; 5 percent **(a)**, 15 percent **(b)**, 25 percent **(c)**, or 35 percent **(d) **of probes with gain.

Importantly, when creating the mimicked data sets we did not generate any simulated ratio values; rather, we formed different selections of values using real experimental data. We believe that this use of real experimental data is of significance for aCGH data. This belief is founded on that, in contrast to expression levels, copy number levels are restricted to a, by comparison, moderate dynamic range. Therefore, when a genomic region is subjected to gain or amplification, the increase of genomic material is relatively substantial. Thus, we reasoned that probes for regions of gain would yield comparably higher average intensities than those for regions of normal copy number and that this, in turn, would result in a correlation between M and A: probes measuring ratios of gain will have higher average intensities. The opposite relationship would apply for probes measuring ratios of loss. Consequently, utilizing normalization strategies based on Lowess would possibly correct for correlations between M and A related to genomic imbalances, resulting in loss of biologically relevant variation. To test this, we subjected the mimicked XXX/XX data sets to Lowess normalization. Once again, as a result of an increased fraction of gain the median M for the X chromosome is shifted, this time from 0.42 to 0.22. The shift can also be observed when looking at autosomes for which the median M is shifted from -0.01 to -0.14 (Figures 2d and 2e). Notably, the variation in M for the X chromosome increases with the fraction of gain (Figure 2d). The interquartile range (IQR) for X chromosome M values increases from 0.21 to 0.24, indicating that Lowess normalization is less suitable when discrete copy number populations exist. When visualizing the normalized data in genome plots the shift, as well as the increase in variation of the X chromosome, is apparent (Figure 2f). To illustrate the differences between how Median and Lowess fail in normalizing the data, and to explain the introduced variation, we created M-A plots for the different data sets including correction lines for the two methods (Figure 3). With an increased fraction of gain the median M value is shifted as seen for the correction line for Median (Figures 3a to d, yellow lines). The correction line for Lowess follows the same shift in the lower range of intensities but diverge at higher intensities (Figures 3a to d, green lines). This divergence indicates that the X chromosome ratios yield higher average intensities and that when the percentage of gain increases an intensity bias is introduced for M. Importantly, this intensity bias is not of a technical nature but represents biologically relevant changes and is a result of inherent properties of aCGH data. In the low range of intensities the Lowess correction line is fitted to local means of M reflecting predominantly autosomes. However, at some point as intensities increase, local means are affected by the X chromosome and then reflect a mixed population of autosome and X chromosome M values, i.e., balanced copy number and gain respectively. As the intensities increase further the locally fitted line will be affected by increasing fractions of X chromosome M values and when normalization is applied this will result in differences in the corrections for X chromosome M values. Thus, this normalization introduces variation. We concluded that Lowess normalization erroneously corrects for biological gain – as gain correlates with intensity in aCGH data – resulting in suppressed ratios and increased variation within copy number populations.

### Normalization of aCGH data using population-based intensity-based lowess

We sought to develop a method that corrects for intensity dependence of M due to technical bias while retaining intensity dependence of biological relevance. We reasoned that if we could stratify aCGH ratios from an experiment with respect to copy number populations, we could use this information to circumvent the drawbacks with Lowess. One way to do this would be to run Lowess on one selected population and then apply the resulting correction line on all M values. We refer to this general strategy of considering copy number populations when using Lowess as population-based intensity-based lowess (popLowess). Applying popLowess would serve two purposes. Firstly, data would be centered at a copy number population rather than a mean or median of a mixture of different and possibly diverse copy number levels. Secondly, correlations between M and A related to technical bias would be identified and corrected for without affecting the intensity dependence due to different copy numbers. To test this strategy, we subjected the mimicked XXX/XX data sets to popLowess. Since we had prior knowledge about this case we could stratify values into copy number populations based on chromosome mapping. All values for autosomes were considered to comprise one population and all values from the X chromosome another.

**Figure 4:**
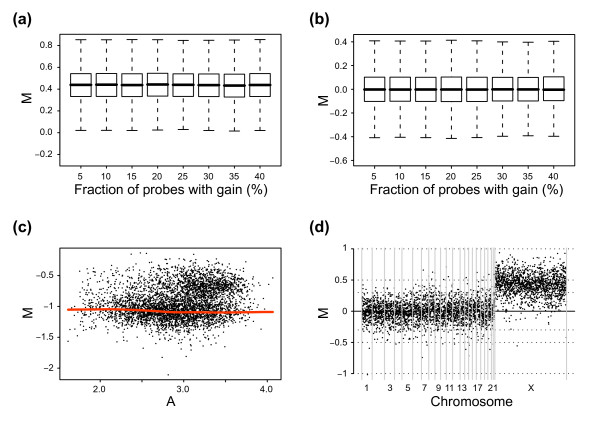
PopLowess normalization of aCGH data. Data from figure 2 is normalized using popLowess. For normalization data was stratified into populations based on genomic mapping of probes. Box-plots display M values after normalization for data sets with varying fraction of probes with gain. **(a) **M values for X-chromosome probes after popLowess normalization. **(b) **M values for autosomal probes after popLowess normalization. **(c) **M-A plot of un-normalized log ratios for the data with 35 percent of probes with gain. Red line corresponds to the popLowess correction line. **(d) **Genome plot after popLowess normalization for the data with 35 percent of probes with gain.

After stratification, raw M and A values for the largest population were used to create a Lowess correction curve. The correction curve was generalized to cover the entire range of A and used to correct all values. Results are presented in figure 4. As expected, no apparent shift in median M or in variation for the X chromosome or autosomes can now be seen between the different percentages of gain (Figures 4a and 4b), demonstrating the effectiveness of popLowess. Notably, the correction line for popLowess exhibits a slight curvature (Figure 4c), indicating that certain intensity dependence of M exists for autosomes, possibly of a technical nature. Albeit small in the presented case, the observed intensity dependence underlines the importance of being able to correct aCGH data for technical bias while retaining biological variation. Based on the results in figure 4, we argue that the strategy behind popLowess offers improved means for normalizing aCGH data. However, we utilized prior knowledge about copy number populations, which guided us in data stratification. Equivalent information for tumor samples can be obtained by karyotyping using, e.g., G-banding, multicolor FISH (M-FISH) analysis, or SKY. This information can also be used to relate a ratio level to an absolute copy number. Having verified copy numbers can guide in centering of data, assuring that gains and losses are presented as relative changes in an appropriate fashion. Then again, these are not trivial experimental procedures and, thus, do not provide a plausible solution in most cases.

### Stratification of M values into copy number populations

**Figure 5:**
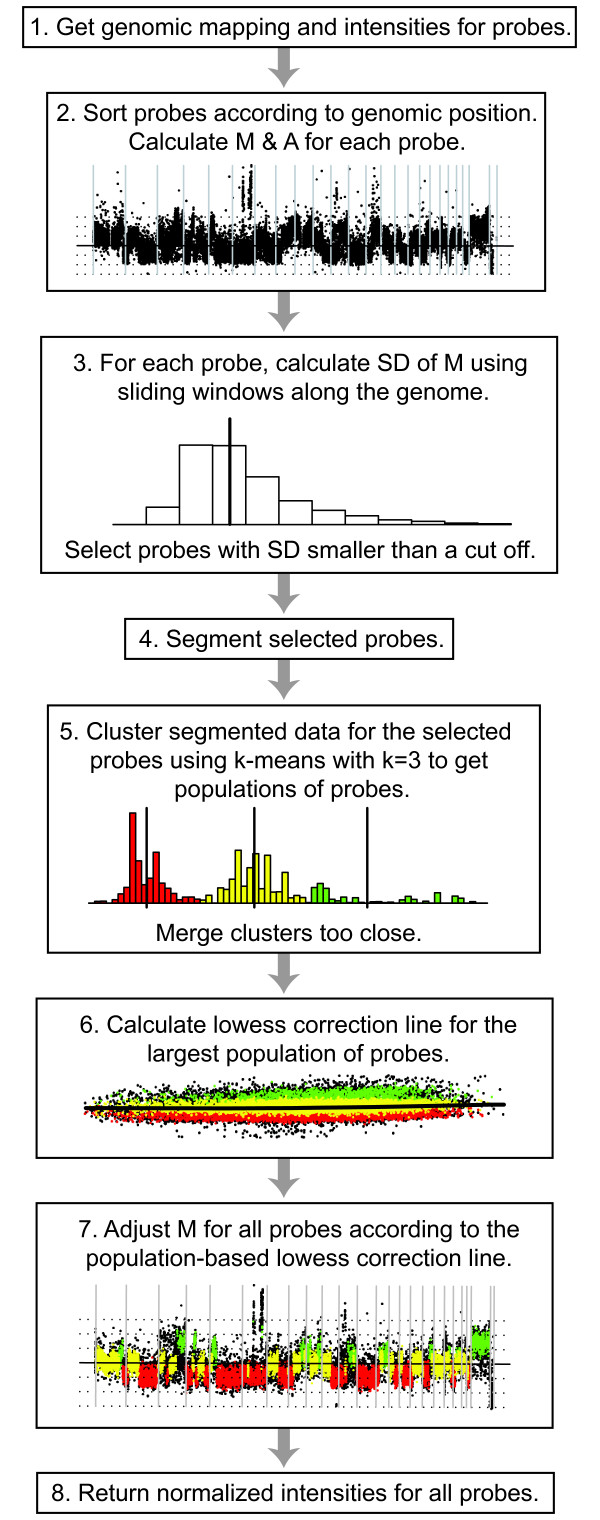
Schematic overview of the proposed popLowess strategy.

**Figure 6:**
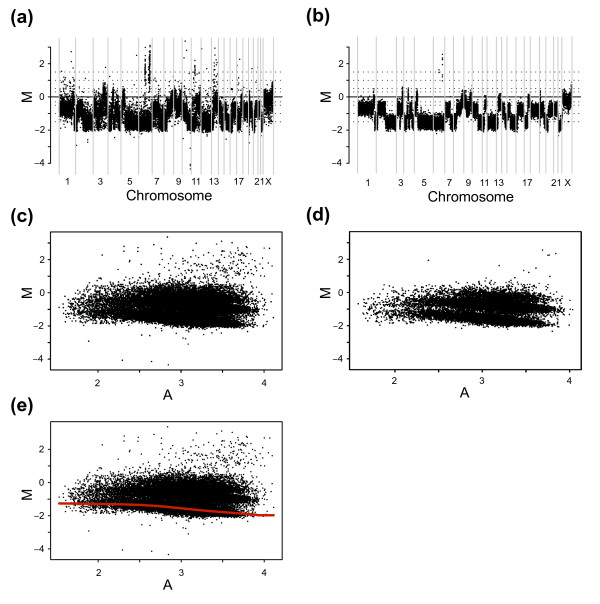
Copy number population enrichment for the L56Br-C1 xenograft analyzed using a tiling 32 K BAC array. **(a) **Genome plot before enrichment. **(b) **Genome plot after enrichment using median of the standard deviation distribution as cut off. **(c) **M-A plot before copy number enrichment. **(d) **M-A plot after copy number enrichment using median of the standard deviation distribution as cut off. **(e)**. M-A plot of all data values with popLowess correction curve superimposed in red.

We aimed at developing a method for stratifying data into populations without prior knowledge regarding copy number allowing us to perform popLowess, and sought to identify populations in an automated fashion that requires minimal manual input and that adapt to varying noise levels. To accomplish this, we took advantage of the naively simplistic form of aCGH data, with a predetermined sequential genomic order of probes, and created a procedure described schematically in figure 5, steps 1–5. By removing outlier data based on ratio similarity between adjacent probes, the proposed strategy enriches populations from genomic regions with similar copy number. That is, regions with high variation in M, e.g., breakpoints or high level amplifications or deletions, are filtered out (Figure 5, steps 1–3). The enrichment of copy number populations can be observed in M-A plots and genome plots displaying data before and after the filter is applied (Figures 6a to 6d). We use a sample adaptive cut off for variation inferred from the data to account for a varying noise level between samples. The filtered data is subsequently segmented to further accentuate the underlying copy number populations and clustered into three distinct groups of values by k-means clustering (k = 3) (Figure 5, steps 4–5). The resulting clusters would roughly correspond to dividing the data into three copy number populations. To address situations where less than three populations exist, a merge cluster criterion can be used to merge clusters with insufficient centre-to-centre distance in M.

To test the performance of our stratification procedure in identifying copy number populations, we used a sample set (data set 8) containing eight hyperdiploid childhood acute lymphoblastic leukemia (ALL) cases previously investigated with aCGH, G-banding and M-FISH [21]. All cases show multiple whole chromosome gains and some cases also minor chromosomal regions of gain. For each case, a population of genomic regions affected by copy number gain was identified based on available karyotyping data. Remaining regions were identified as a diploid population. We performed steps 1–5 of the popLowess stratification procedure on each case using a merge cluster criterion of M = 0.3. Effectively, two popLowess populations were obtained for each case, corresponding closely to the karyotyping data of a normal diploid population and a population of copy number gain. For both the gain and diploid popLowess populations the total number of called probes divided by expected total number of probes from karyotyping data was calculated. Furthermore, the fraction of correctly called probes by popLowess for the specific regions of gain defined by karyotyping was calculated. The results demonstrate that the procedure can effectively stratify data into enriched populations that represents discrete copy number levels (Table 1).

**Table 1:** Comparison of popLowess enriched population assignment to karyotyping data for eight hyperdiploid cases [21]

Case	Gain (called/karyotype)*	Diploid (called/karyotype)**	Gain (fraction of karyotype called)***
1	0.97	1.05	0.99
2	0.87	1.09	1.00
3	1.00	1.00	0.85
4	1.14	0.88	0.85
5	0.89	1.21	0.99
6	0.97	1.08	1.00
7	0.95	1.11	0.80
8	1.09	0.76	0.63

Two populations corresponding to a normal diploid population and a population of copy number gain were created using steps 1–5 of the procedure in figure 5. The ratio of probes called as belonging to the gain population and probes determined as gain according to karyotype data is shown*. The equivalent ratio for the diploid population is also shown**. In addition, the fraction of probes called correctly as gain within regions determined as gain by karyotype data is displayed***.

### A procedure for normalization of aCGH data using popLowess

Once data is stratified into sets of enriched copy number populations we can select one, e.g., the largest, to perform Lowess normalization on. The generated correction curve must be generalized to cover the full range of A allowing for correction of all M values (Figure 6e). This procedure will ensure that the lowess derived correction line trails one population and remains unaffected by adjacent ones. We refer to this action as popLowess-o (where the letter *o *is a mnemonic for *one*) as it makes use of one population to derive a correction line for all data. The complete procedure of data stratification and popLowess normalization is shown in figure 5, steps 1–8. Naturally, once data is stratified alternative variants of calculating normalization corrections are imaginable. For example, one could fit lowess lines to each population and correct them individually or one could individually center populations and then use the combined data to create a lowess derived correction line. We refer to these alternatives as popLowess-i (where the letter *i *is a mnemonic for *individual*) and popLowess-c (where the letter *c *is a mnemonic for *common*) respectively. The latter alternative has the added advantage of reducing the degree to which the correction line needs to be extrapolated to cover the full range of A. Both alternatives require an additional step to center a selected copy number population at M = 0. The variants popLowess-o and popLowess-c rely on that the intensity-based curvature in M-A space is reasonably shared between populations.

### Selecting a population to represent intrinsic copy number

The normalization procedure presented herein will center a population with unknown copy number at M = 0. The rationale for selecting an appropriate population for this purpose can differ depending on samples analyzed and the aim of a project. For instance, in the field of cytogenetics, gains and losses in tumors are by convention described as net changes relative to intrinsic balanced copy number, i.e., relative ploididy. As the number of centromeres determines ploidity, a parallel rationale would be to relate imbalances relative to the largest identified population and therefore center this population at M = 0. However, in some applications it might be more appropriate to relate imbalances to a normal diploid state. Thus, selecting a population to center data at can include using prior knowledge about regions with known copy number or selecting the middle population out of three, if present. Irrespectively of preferences of how data best be centered, the proposed popLowess procedure will alleviate the normalization problems related to mixed copy number populations. Importantly, when performing focused aCGH with specialized arrays that do not cover the entire genome, or comprise probes with a disproportioned focus on specific genomic regions, even CNAs that affect a minor part of the genome can introduce a significant correlation between copy number and intensity, and can result in misinterpretations of how a given ratio level relate to copy number.

### Application to tumor specimens on different aCGH platforms

**Figure 7:**
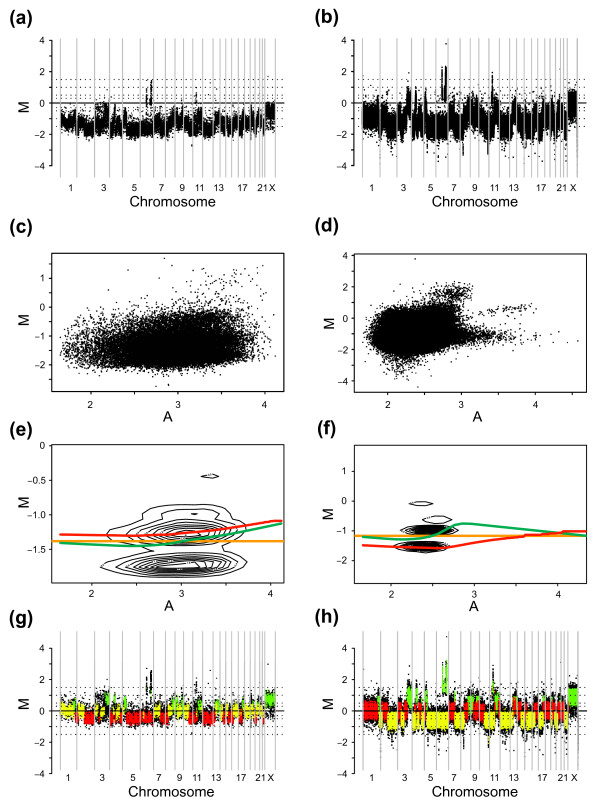
*BRCA1 *mutation positive breast cancer sample analyzed using a tiling 32 K BAC array and an Agilent 244 K oligonucleotide CGH array. Correction lines for Median (orange), Lowess (green), and popLowess (red) normalization are superimposed in panels (e) and (f). Identified copy number populations are differentially colored in panels (g) and (h) according to size where yellow corresponds to the largest identified copy number population, red to the second largest, and green to the smallest. Data in panels (g) and (h) are centered on the middle population **(a) **Genome plot of un-normalized BAC data. **(b) **Genome plot of un-normalized Agilent data. **(c) **M-A plot of un-normalized BAC data. **(d) **M-A plot of un-normalized Agilent data. **(e) **Contour plot of copy number population enriched BAC data. **(f) **Contour plot of copy number population enriched Agilent data. **(g) **Genome plot of BAC data after popLowess. **(h) **Genome plot of Agilent data after popLowess.

We next set out to test the proposed popLowess strategy on tumor aCGH data that display a more complex pattern of genomic imbalances and to test its performance on data derived from different array platforms. Figures 7a and 7c show genome- and M-A plots of a primary *BRCA1 *mutation positive breast cancer analyzed on a tiling 32 K BAC array. The genomic profile (Figure 7a) shows clear regions of aberration; however factors such as normal cell contamination and potential tumor heterogeneity have decreased the range in M for the sample specific CNAs. In the M-A plot (Figure 7c) the different copy number populations are not as distinct as for the sample in figure 6, likely making it more difficult to identify the copy number populations. We used the proposed popLowess strategy to identify copy number populations and visualized the result in a contour plot. Results are shown in figure 7e together with correction lines for Median, Lowess and popLowess. As observed in figure 7e neither the Lowess, nor the Median correction curve, accurately track a single copy number population. Figure 7g shows the normalized genomic profile after popLowess with the identified populations colored. The genomic profile has now been centered correctly and matches a previous report with detailed investigations of this tumor [19]. Figure 7 (panels b, d, f and h) show the same collection of plots for the same tumor profiled using Agilent 244 K CGH oligonucleotide arrays (DLR-value 0.196). The vast number of probes and a considerably higher level of technical noise for raw data, renders it virtually impossible to visually distinguish the populations, clearly seen in the genomic profile (Figure 7b), using the 2D M-A plot (Figure 7d). Employment of the popLowess strategy enriches copy number populations of data as observed in the contour plot (Figure 7f). Similarly to the BAC array case, neither the Median, nor the Lowess correction curve, accurately track a single copy number population. Figure 7h shows the genomic profile after popLowess with identified populations colored. To assess the effect of normalization on variation for data in Figure 7 we calculated IQR for M values of identified populations. In the BAC case the average change in IQR for the three identified populations was an increase by 0.0012 when Lowess normalization was applied. Contrary, after popLowess the average change in IQR was a decrease by -0.00029. For the Agilent case the corresponding changes were an average increase by 0.059 after Lowess compared to an average decrease by -0.0011 after popLowess. Again, we conclude that Lowess, by not tracking a single population, erroneously corrects for CNAs resulting in an increased variation within copy number populations.

**Figure 8:**
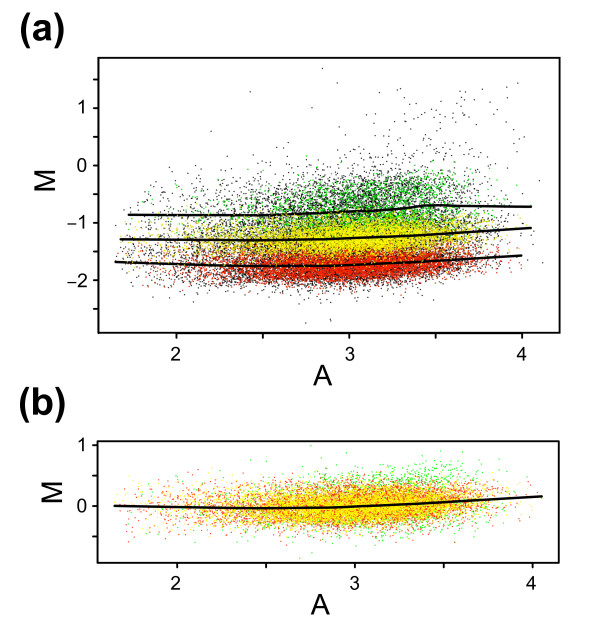
M-A plots of popLowess normalization variants for BAC data. Data from figure 7 is used. **(a) **M-A plot with lowess correction lines for each identified population superimposed. **(b) **M-A plot of median centered populations (popLowess-c) with lowess correction line based on all populations.

In order to illustrate the differences between alternative popLowess strategies we used variants to derive correction lines (Figure 8). In figure 8a correction lines for individual populations are presented. The popLowess strategy (popLowess-o) used to produce the results in figure 7 corresponds to normalizing data by selecting one of the correction lines in figure 8a. For the results in figure 7, the correction line for the largest population was selected (colored yellow in Figures 7 and 8). In figure 8b the correction line derived from popLowess-c is shown together with individually median centered populations. As mentioned, popLowess rely on that the intensity-based curvature in M-A space is reasonably shared between populations. When inspecting the individual correction lines in figure 8a, populations appear to display similar intensity-based curvature although small differences appear. Differences may partly be a result of extrapolating correction curves at the ends. A thorough investigation of these differences, although outside the scope of this study, would be of interest.

### Comparison of popLowess strategy to standard normalization methods

We set out to test if the popLowess strategy could systematically reduce variation in M within copy number populations in different aCGH data sets. We hypothesized that when correction curves cross, or not accurately track, copy number populations; or when intensity-based curvature is not properly addressed, a larger variation in M is obtained after normalization. To this aim, we compared the performance of the popLowess strategy versus Median and Lowess using seven different aCGH data sets (data sets 1–6, 8). The data sets cover three different types of aCGH platforms hybridized with a variety of cell line and tumor samples displaying a large variation of CNAs.

We used the strategy in figure 5 to identify copy number populations in each of the data sets. We then normalized each data set in parallel using popLowess, Lowess, and Median. After normalization, we calculated standard deviations of M for each identified population for each method and compared results.

Results from the comparison are displayed in table 2, showing that the popLowess strategy generated normalized copy number data with smaller standard deviations in M within identified populations for all comparisons and data sets. We repeated the test using the inter-quartile range (IQR) of M for each population instead of the standard deviation and obtained similar results (data not shown).

**Table 2:** Comparison of effect on population variance between different normalization strategies

	P-values for data sets							
		
	Data set	1 [23]	2 [25]	3 [20]	4 [8]	5	6	8 [21]
								
	Nbr of samples	7	28	10	52	8	8	8
	Platform	BAC 32 K	BAC 32 K	BAC 32 K	BAC 1 Mb	Agilent 244 K	Agilent 44 K	BAC 32 K
popLowess vs Lowess	All populations	1.1e-4	7.0e-12	5.6e-8	3.4e-28	2.5e-05	1.6e-4	7.2e-5
	Population 1	7.8e-3	1.4e-5	9.8e-4	9.9e-32	2.0e-3	2.0e-3	3.9e-3
	Population 2	7.8e-3	1.5e-6	9.8e-4	7.4e-4	2.0e-3	0.09	3.5e-2
	Population 3	0.23	6.3e-3	2.0e-2	2.5e-7	0.25	0.09	0.25

popLowess vs Median	All populations	< 1e-32	< 1e-32	< 1e-32	< 1e-32	< 1e-32	< 1e-32	< 1e-32
	Population 1	7.8e-3	3.7e-9	9.8e-4	9.9e-32	2.0e-3	2.0e-3	3.9e-3
	Population 2	6.3e-2	1.4e-5	0.17	3.8e-2	0.50	0.09	0.14
	Population 3	0.23	9.0e-5	0.25	0.28	0.25	0.50	0.75

P-values for different populations for data sets are shown. The test corresponds to the null hypothesis that lower standard deviations for popLowess are obtained by chance. Population 1 always relates to the largest identified population (# probes), population 2 to the second largest and population 3 to the smallest.

Since we do not have prior knowledge of CNAs in most of the cases we cannot evaluate variation within confirmed genomic regions of similar copy number. Therefore, one could argue that the better performance of popLowess, resulting in lower variation within populations when compared with conventional normalization, is biased by the fact that populations are inferred from the data. However, from looking at the data in table 1, and at the genome plots in figure 7 (panel g and h) we note that the identified populations reflect regions with discrete copy number levels. Therefore, we argue that decreased intra population variation is beneficial to both interpretation and downstream analysis and provides improved data quality.

### Spatial effects

Presence of technical artifacts in array data resulting in correlation between M and spatial probe location on the array is a well-known and previously described phenomenon. We focused on two plausible consequences of such spatial effects in aCGH data. Firstly, affected values can introduce populations that compromise normalization in the same way as copy number populations. Secondly, affected values will be incorrectly scaled compared to non-affected.

We reasoned that ratios biased by spatial artifacts are controlled for by our proposed popLowess strategy as it filters outlier data guided by genomic mapping. Thus, when calculating an intensity dependent correction for normalization, our strategy would not be compromised by spatial bias as affected values are disregarded together with values from break points, high-level amplifications, and homozygous deletions. On the other hand, popLowess does not correct for spatial effects and affected values would remain incorrectly scaled after normalization even if the intensity bias is removed.

As the proposed popLowess strategy does not correct for spatial effects, we reasoned that a pre-normalization step might be appropriate for data displaying spatially related bias in order to properly scale affected values. This could be accomplished by applying one of many available spatial correction methods [15-17], or variations thereof, prior to popLowess. However, since we have shown that genomic imbalances correlate with intensity, we are cautious about addressing spatial effects using pre-normalization algorithms that are intensity-based.

To test our reasoning we applied popLowess to data set 7. Samples in this set have little to no genomic alterations but the data display variation in M-A curvature and spatial effects. Data set 7 was normalized using popLowess, block-based Median followed by popLowess, or block-based Lowess followed by popLowess. For popLowess, by itself or in combination with a pre-normalization step, a merge cluster criteria of 0.3 in M was employed to account for the presence of only two copy number populations.

As a measurement of spatial effects we calculated the standard deviation of medians of M from pin-tip blocks before and after normalization. We found that spatial bias may be corrected for by a pre-normalization step, preceding popLowess (Table 3).

**Table 3:** Effect of pre-normalization to correct spatial bias prior to applying popLowess

	un-normalized*	popLowess**	pre-normalization by block-based Median***	pre-normalization by block-based Lowess****
XY vs XY	0.062	0.062	0.009	0.003
XY vs XY	0.067	0.067	0.003	0.003
XX vs XX	0.097	0.069	0.035	0.003
XX vs XX	0.125	0.127	0.031	0.004
XXX vs XX	0.051	0.049	0.003	0.004
XX vs XY	0.046	0.045	0.003	0.003
XXXX vs XX	0.060	0.033	0.004	0.004
XXX vs XY	0.058	0.058	0.007	0.005
XXXX vs XY	0.060	0.058	0.008	0.004

Standard deviation of medians of M from pin-tip blocks. Standard deviation is calculated before normalization* and after normalization using popLowess alone** or together with a pre-normalization step. Applied pre-normalization steps include either block-based Median*** or block-based Lowess****.

We conclude that the proposed popLowess strategy is robust in the sense that it can handle the presence of otherwise deleterious populations without relying on them. We also conclude that, whereas popLowess is inert to spatial effects, in the sense that it does not compromise calculation of an intensity dependent correction, a pre-normalization step that correct for spatial bias is warranted.

### Adaptive sample-specific thresholds for calling copy number change

During development of the popLowess strategy, we recognized that the sample-specific cut-off value (Figure 5, step 3) could be used to assess noise level in data and to assign thresholds for copy number imbalances on a sample-specific basis. Several reports [5,8,22,23] have utilized global thresholds in M for calling CNA as gains or losses. These thresholds are assigned by adding/subtracting a value in M from a base line typically at M = 0. Determining suitable thresholds may be problematic in large sample sets with samples of varying quality and heterogeneity, often the case for tumor studies [10], and may result in setting too conservative thresholds for certain samples in order to avoid erroneous CNA calls. Deriving sample-specific threshold values scalable for desired stringency in an automated fashion is then of relevance.

A parallel can be made to the derivative log ratio spread (DLR) value calculated by the Agilent CGH Analytics software. The DLR-value can be used to assess hybridization quality and provide a sample scalable threshold for calling CNAs using, e.g., the Z-scoring algorithm in the CGH Analytics software.

**Figure 9:**
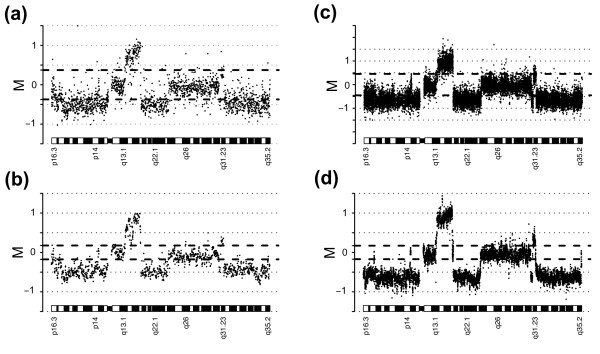
Use of sample adaptive gain/loss thresholds. Thresholds are applied to data from figure 7. **(a) **Copy number profile derived from BAC data for chromosome 4 after popLowess normalization with adaptive thresholds superimposed (± 0.372). **(b) **Copy number profile for chromosome 4 for data from panel (a) after smoothing (250 kBp) and new threshold estimate (± 0.176). **(c) **Copy number profile of Agilent data for chromosome 4 after popLowess normalization with adaptive thresholds superimposed (± 0.453). **(d) **Copy number profile for chromosome 4 for data from panel (c) after smoothing (50 kBp) and new threshold estimate (± 0.171).

We used sample specific level thresholds derived from popLowess on aCGH data for a *BRCA1 *mutation positive tumor analyzed on two array platforms (Figure 9). Figure 9a shows thresholds after popLowess normalization for the BAC array data and figure 9b after application of a 250 kBp smoothing window. Figure 9c shows the same tumor analyzed on the Agilent platform after popLowess and figure 9d after application of a 50 kBp smoothing window. As shown in figure 9, thresholds are automatically adapted to specifically match data. We believe that the use of sample-specific adaptive thresholds will greatly facilitate the analysis of larger aCGH data sets that include samples of varying heterogeneity and quality.

### Normalization affects downstream analysis

**Figure 10:**
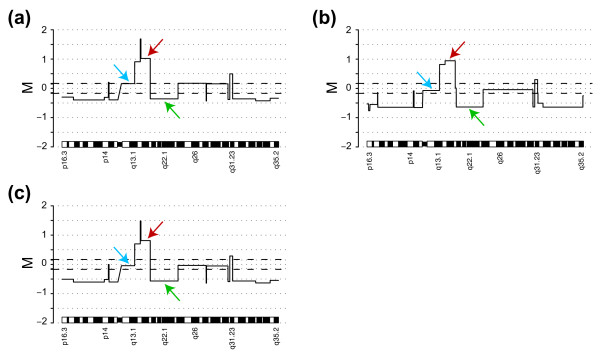
Example of segmentation of data after alternative normalization methods. Segmented copy number profile of chromosome 4 from smoothed (50 kBp) Agilent data for the sample from figure 7 with superimposed adaptive thresholds (± 0.171). Three different segments are highlighted with colored arrows in each panel to exemplify regions with different copy number level **(a) **Segmentation applied after Lowess. M values for selected segments; blue arrow 0.16, red arrow 1.02, and green arrow -0.36. **(b) **Segmentation applied after popLowess. M values for selected segments; blue arrow -0.07, red arrow 0.94, and green arrow -0.64.**(c) **Segmentation applied after Lowess normalization subsequently followed by centralization of data on median M of an individual population. M values for selected segments; blue arrow -0.05, red arrow 0.81, and green arrow -0.57.

To exemplify how normalization can affect downstream analysis and interpretation we used data generated from the Agilent array presented in Figure 7. We normalized the raw data shown in Figure 7 (panels b and d) with either Lowess or popLowess. Correction lines for both normalization methods are shown in Figure 7f. We then smoothed data (50 kBp window) and performed segmentation using the CGHplotter algorithm [14]. Results for chromosome 4 are shown in Figure 10. In the given example, segmentation after Lowess and popLowess (Figures 10a and 10b respectively) broadly identifies the same break points. However, after Lowess the data is not centered on any of the identified segments as a result of the correction line not tracking a specific population in the raw data (Figure 10a). Contrary, after popLowess the data is centered on a specific segment level (Figure 10b; e.g. blue arrow). Shifting data to center it on a specific population can be done after any conventional normalization method. An example is shown in Figure 10c where data have been centered after Lowess. Determining the point at which data is centered can for example be achieved by stratifying data into populations using the method presented herein or by the method proposed by Lipson et al. [24]. Importantly, to center data after Lowess does not alleviate the aforementioned problem of introduction of variation and the inappropriate correction of biological gain and loss. As a result, in the example given the dynamic range between segments of gain (Figure 10, red arrows) and loss (Figure 10, green arrows) is reduced after Lowess compared with popLowess, 1.38 versus 1.58. For Lowess a smaller dynamic range between levels is present in both directions relative to the baseline level (Figure 10). Reduced dynamic range or inappropriate centralization of aCGH data can result in misinterpretations when investigating genomic copy number profiles.

## Conclusion

We show that the presence of copy number populations in aCGH data deleteriously affects normalization using curve-generating algorithms such as intensity-based lowess and may cause erroneous centering of data. We demonstrate that genomic imbalances correlate with intensity in aCGH data and therefore must be accounted for during normalization in order to correct for intensity dependence of M due to technical bias while retaining intensity dependence of biological relevance. Here we propose a population-based normalization strategy that accounts for the presence of copy number populations. We show that benefits of a population-based normalization approach are clearly recognized for data displaying numerous CNAs. We also demonstrate that the proposed procedure can be applied to assign adaptive sample-specific thresholds for calling copy number changes. We appreciate that the suggested strategy represents only one conceivable way of implementing population-based normalization and that any implementation that effectively discerns copy number populations in aCGH data, whether utilizing prior knowledge regarding samples or inference from the data itself, could be used. In addition, once copy number populations are identified, this information can be used in a variety of ways to circumvent highlighted problems related to conventional normalization of aCGH data. Taken together, we demonstrate that copy number populations in aCGH data should be accounted for during normalization and that the proposed normalization strategy, as well as the adaptive sample-specific level scaling, provides powerful and convenient means for improved copy number analysis using aCGH.

## Methods

### Data sets

We used eight data sets derived from BAC arrays and from Agilent 244 K oligonucleotide CGH arrays to evaluate normalization methods. Data set 1 consists of seven breast cancer cell lines analyzed using tiling 32 K BAC arrays [23]. Data set 2 consists of 28 lung cancer cell lines analyzed using tiling 32 K BAC arrays [25]. Data set 3 consists of ten breast cancer cell lines analyzed using tiling 32 K BAC arrays [20]. Data set 4 consists of 52 breast cancer tumors analyzed in dye-swaps on 1 Mb BAC arrays [8]. Data set 5 consists of 8 breast cancer tumors and one dye-swap analyzed using Agilent 244 K oligonucleotide CGH arrays [26]. These tumors displayed DLR values between 0.196 and 0.364 when analyzed with Agilent CGHAnalytics software ver 3.4.27 [26]. Data set 6 was created from data set 5 by matching the oligonucleotide probe IDs from the 244 K arrays to the Agilent 44B probe IDs available through Agilent eArray [27], thus creating a virtual 44 K oligonucleotide CGH array. Of 42,447 genome-mapped probe IDs on the 44B array, 41,599 were found on the 244 K arrays (98%). Data set 7 consists of nine hybridizations of chromosome X aberrant cell lines with karyotype 47, XXX and 48, XXXX, and male 46, XY and female 46, XX samples in various combinations [20]. Samples in data set 7 are expected to display a normal karyotype for chromosomes 1–22. Data set 8 consists of eight hyperdiploid childhood ALL cases analyzed using tiling 32 K BAC arrays [21].

### Pre-filtering and conventional normalization of aCGH data

All data sets were loaded into BioArray Software Environment (BASE) [28] for analysis. Positive and non-saturated spots were background corrected using the median foreground minus the median background signal intensity for each channel and log ratios (M) were calculated from the background corrected intensities. In all analysis we used M = log_2_(int1/int2) and A = log_10_(sqrt(int1*int2)), where int1 and int2 are background corrected intensities from the investigated sample and reference, respectively. Data sets 1–4 and 7–8 were filtered for signal-to-noise ratio for each spot in both channels according to published reports and the remaining data sets for signal-to-noise ratio > 5 in both channels before BASE implemented software plug-ins of the different normalization strategies were employed. A lowess smooth factor of 0.33, delta of 0.1, and four iterations were used for standard Lowess, popLowess and block-based lowess normalization. Block group size was set to 1 for all block-based normalizations.

### Population-based intensity-based lowess

A schematic overview of the proposed popLowess normalization strategy is shown in figure 5. The approach is applied on a per sample basis starting with genomic mapping and raw intensities (int1 and int2) for N probe IDs (step 1, Figure 5). The probes are sorted according to genomic position and M and A are calculated for each probe (step 2, Figure 5). Next, a standard deviation in M is calculated for each probe in sliding windows of user-defined size along the genome. The resulting distribution of N standard deviations is subjected to a cut-off criterion generating K probes with standard deviations < cut-off for continued population analysis (step 3, Figure 5). A moving window size of 11 probes was used and the median of the standard deviation distribution was used as cut-off value. This selection criterion is sample adaptive avoiding problems with using a global cut-off criterion. The K selected probes are next segmented on a per chromosome basis using, e.g., the CGHplotter algorithm [14] or the faster circular binary segmentation (CBS) algorithm [13] (step 4, Figure 5). Herein, the segmentation algorithm proposed by Autio et al. was used with the constant for computing the number of changes (c-parameter) set to 10 [14]. Segmented values are used to cluster the K probes into three distinct clusters by means of robust k-means clustering (step 5, Figure 5). After clustering, there is an option to merge clusters with cluster centers close to each other. Merging is typically useful for samples not displaying three populations, e.g., samples with 1 or 2 copy number populations. When indicated, a merge cluster criterion of 0.2 or 0.3 in M was used. The resulting data consists of 1–3 distinct populations of data that contains information about the genomic mapping, M, and A for each probe. The largest population is selected for lowess normalization [29] generating a population specific correction curve (step 6, Figure 5). The correction curve is next extrapolated to the entire range of A and used to correct M for all N reporters similar to Lowess (step 7, Figure 5). The extrapolation is done conservatively in the end points of A by using the first/last data point of the population specific correction curve to level out the global correction curve horizontally in the M-A plot thereby moderating the impact of extreme points or missing values. After lowess correction, one population is selected as the center population and all data is shifted such that this population obtains median M equal to 0. Selection of a center population can be based on different assumptions. Finally, the normalized int1 and int2 intensities are returned (step 8, Figure 5). By not segmenting the entire set of observations, and by setting the crucial segmentation parameters for detecting breakpoints in the lower scale, speed is gained while still retaining robustness as long as the standard deviation cut off is not set too low. The purpose of segmentation is to refine large regions with identical copy number and not to detect small complex copy number alterations.

### Comparison of normalization methods

For comparisons, the R implemented lowess function was used to create lowess-normalized data. For each identified population (step 1–5, Figure 5) in every sample in data sets 1–6 and 8, the standard deviations in M of the reporters in the population after Lowess, popLowess, and no normalization (equal to Median) were calculated separately. The number of populations in a data set for which the popLowess strategy rendered a lower standard deviation compared to the competitor was calculated. To evaluate if popLowess resulted in a significant number of populations with lower standard deviations, one sided p-values were calculated using the binomial distribution with p = 0.5. This binomial test corresponds to the null hypothesis that lower standard deviations for popLowess are obtained by chance. This comparison was done both when studying all populations as a whole and for each population individually.

### Sample adaptive gain/loss thresholds

Sample adaptive thresholds for calling gain or loss can be generated by performing steps 1–3 in Figure 5 using the same form of data input and standard deviation cut-off criteria. The identified standard deviation cut-off value can be scaled by multiplicative factors to generate sample specific gain/loss thresholds of desired stringency for downstream applications, e.g., calling CNAs after segmentation. Before creating sample adaptive thresholds, data was pre-filtered and normalized using the popLowess strategy. Sample adaptive thresholds for the Ca13928 breast tumor were created before and after a smoothing window of 250 kBp size for 32 K BAC data and 50 kBp for Agilent 244 K data. Thresholds were estimated using a chromosomal moving window of size 1% of the total probe number for each chromosome separately and the standard deviation cut-off value was selected as the median of the standard deviation distribution. The cut-off value was scaled by a factor 2 to create the ± thresholds in M displayed in figure 9.

### Availability and requirements

An implementation of popLowess in R  is available both as a plugin to the BioArray Software Environment (BASE) [28] and as a stand-alone version.

Project name: popLowess

Project home page: 

Operating system(s): Platform independent

Programming language: R

License: GNU GPL

## List of abbreviations

aCGH: array-based CGH

ALL: acute lymphoblastic leukemia

BAC: bacterial artificial chromosome

BASE: BioArray Software Environment

CGH: comparative genomic hybridization

CNA: copy number aberration

CNV: copy number variation

FISH: Fluorescence in situ hybridization

IQR: Inter Quartile Range

Lowess: Global intensity-based lowess normalization

Median: Global median normalization

popLowess: population-based intensity-based lowess normalization

SKY: Spectral karyotyping technique

## Competing interests

The author(s) declares that there are no competing interests.

## Authors' contributions

All authors participated in the development of the model. JS implemented and developed the methods. JS and MR performed the statistical tests. JVC conceived the study. JS and JVC drafted the manuscript. All authors participated in the design of the study and in completing the manuscript. All authors read and approved the final manuscript.
